# Refractive outcomes among glaucoma patients undergoing phacoemulsification cataract extraction with and without Kahook Dual Blade goniotomy

**DOI:** 10.1186/s40662-019-0153-2

**Published:** 2019-09-19

**Authors:** Erin G. Sieck, Cara E. Capitena Young, Rebecca S. Epstein, Jeffrey R. SooHoo, Mina B. Pantcheva, Jennifer L. Patnaik, Anne M. Lynch, Malik Y. Kahook, Leonard K. Seibold

**Affiliations:** Department of Ophthalmology, University of Colorado School of Medicine, 1675 Aurora Court F731, Aurora, CO 80045 USA

**Keywords:** Kahook Dual Blade, goniotomy, refractive surprise, glaucoma, cataract

## Abstract

**Background:**

Glaucoma patients undergoing phacoemulsification alone have a higher rate of refractive surprise compared to patients without glaucoma. This risk is further increased with combined filtering procedures. Indeed, there are few and conflicting reports on the effect of combined phacoemulsification and micro-invasive glaucoma surgery (MIGS). Here, we look at refractive outcomes of glaucoma patients undergoing phacoemulsification with and without Kahook Dual Blade (KDB) goniotomy.

**Methods:**

Retrospective chart review of 385 glaucomatous eyes of 281 patients, which underwent either phacoemulsification alone (*n* = 309) or phacoemulsification with KDB goniotomy (*n* = 76, phaco-KDB) at the University of Colorado. The main outcome was refractive surprise defined as the difference in target and postoperative refraction spherical equivalent greater than ±0.5 Diopter (D).

**Results:**

Refractive surprise greater than ±0.5 D occurred in 26.3% of eyes in the phaco-KDB group and 36.2% in the phacoemulsification group (*p* = 0.11). Refractive surprise greater than ±1.0 D occurred in 6.6% for the phaco-KDB group and 9.7% for the phacoemulsification group (*p* = 0.08). There was no significant difference in risk of refractive surprise when pre-operative IOP, axial length, keratometry or performance of KDB goniotomy were assessed in univariate analyses.

**Conclusion:**

There was no difference between refractive outcomes of glaucomatous patients undergoing phacoemulsification with or without KDB goniotomy.

## Background

Phacoemulsification has become the standard of care for cataract removal in the United States and remains one of the most cost-effective treatments in the medical industry [1, 2]. It is predicted that by 2020, more than 30 million people worldwide will undergo cataract removal annually [3]. Surgical techniques, instrumentation and intraocular lens (IOL) options within the field are constantly advancing to further improve outcomes. Along with these advances, patient expectations have grown, driving an increased demand for predictable refractive outcomes in patients with and without glaucoma. Novel IOL formulas have been introduced in recent years and have improved refractive accuracy in the general population [4, 5]. These calculations however, are less accurate in patients with both open and closed angle glaucoma [6, 7]. Furthermore, a higher rate of refractive surprise has been shown in glaucoma patients undergoing phacoemulsification [8].

Refractive outcomes for glaucoma patients can be even more difficult to predict when phacoemulsification is combined with glaucoma surgery. Patients undergoing filtering surgery at the time of cataract removal were shown to experience a higher rate of refractive surprise and induced cylinder [9]. Even in patients with a prior history of filtering surgery there is a higher rate of refractive surprise, though several other groups have not been able to validate these findings [10–13].

More recently, micro-invasive glaucoma surgery (MIGS) and less invasive ab interno procedures to treat glaucoma have gained popularity as an adjunct procedure during cataract removal. These procedures typically utilize the same clear corneal incisions of traditional phacoemulsification and do not produce a sub-conjunctival filtering bleb. One potential advantage of these less invasive approaches compared to traditional filtering surgery is a reduced risk of inducing a significant refractive surprise. However, to date, there is only preliminary data regarding their impact on refractive outcomes. Luebke et al. demonstrated no difference in refractive outcomes between patients undergoing combined trabectome-cataract surgery compared to cataract surgery alone [14]. Manoharan et al. demonstrated there was no difference in refractive outcomes in glaucoma patients who underwent combined phacoemulsification with iStent compared to phacoemulsification alone [8]. Moreover, there have been conflicting results on the impact of endocyclophotocoagulation (ECP) at the time of phacoemulsification and its influences on refractive outcomes [15–17].

The Kahook Dual Blade (KDB, New World Medical, Rancho Cucamonga, CA) is a novel goniotomy device used for excising a strip of trabecular meshwork to lower intraocular pressure (IOP) [18]. Currently, many studies have demonstrated that when used with or without phacoemulsification, KDB goniotomy produces a significant decrease in intraocular pressure and medication burden with no adverse effect on post-operative visual acuity [19–21]. However, as mentioned, few studies have evaluated whether a risk of refractive surprise exists in angle-based procedures. To our knowledge, no published study has specifically examined whether an increased risk of refractive surprise occurs after KDB goniotomy. In this present study, we retrospectively evaluated the refractive outcomes of glaucoma patients who underwent phacoemulsification with and without KDB goniotomy.

## Methods

The University of Colorado Department of Ophthalmology’s Cataract Outcomes Registry was used to identify patients for inclusion in this retrospective cohort study. All cataract surgeries performed at the University of Colorado Health Eye Center are included in this database. The Colorado Multiple Institutional Review Board approved the study. A retrospective chart review of glaucomatous patients who underwent either phacoemulsification alone or phacoemulsification with KDB goniotomy (phaco-KDB) from January 1, 2014 to December 31, 2016 was performed. The comparison group of patients with glaucoma undergoing phacoemulsification alone had their surgeries performed by both glaucoma-trained and cornea-trained surgeons at our institution. The second group undergoing both phacoemulsification and goniotomy were performed by one of four fellowship trained glaucoma specialists. The database included 920 eyes undergoing cataract surgery with the prior diagnosis of glaucoma. Eyes were excluded if they had a traumatic cataract (*n* = 4) or cataract surgery was combined with a vitrectomy procedure (*n* = 24). Additionally, eyes were excluded if the cataract surgery was combined with a filtering procedure (*n* = 18) or the eye had prior refractive surgery (*n* = 36). Cases were excluded if they were combined with endoscopic cyclophotocoagulation (ECP; *n* = 308), Trabectome (*n* = 1), bleb or drainage device revision (*n* = 3), or corneal transplant (*n* = 2). Patients without a stated pre-operative target or post-operative refraction were also excluded (*n* = 139). Intraocular lens power calculations were performed using partial coherence interferometry (IOLMaster 500, Carl Zeiss Meditec AG) with immersion ultrasound supplementation as needed. The lens formulas used were according to surgeon preference and generally as follows: Hoffer Q was used for axial lengths (ALs) shorter than 23.0 mm, Holladay 1 for ALs between 23.0 mm and 26.0 mm, and SRK/T for 26.0 mm and longer.

Data were collected on patient demographics, ocular characteristics, pre-operative refractive target, post-operative refraction, subtype of glaucoma, visual acuity and pre- and post-operative IOP. Visual acuity was assessed via Snellen chart both pre-operatively and post-operatively. The Snellen chart visual acuity was converted to equivalent logMAR notation [22]. Pre-operative refractive target was defined as the intended target refraction based on the IOL Master 500 calculations with ultimate lens selection at the discretion of the surgeon. The main outcome measure was the final post-operative refraction compared to the pre-operative target and the presence of a refractive surprise. When multiple refractions were taken, the refraction with the best visual acuity was used or an average of refractions if visual acuities were equivalent. Refractive surprise was defined as a difference in target and post-operative refraction spherical equivalent greater than ±0.5 diopters (D) or ± 1.0 D.

### Statistical analysis

Logistic regression modeling with generalized estimating equations used to account for correlation between eyes since a person could have two eyes included in the database. Predictors of refractive surprise greater than ±1.0 D and associations between covariates and KDB were assessed with univariate logistic regression modeling. The final multivariable model of predictors for refractive surprise included KDB as the main explanatory variable and covariates with *p* < 0.10 in univariate analysis.

## Results

A total of 385 glaucomatous eyes of 281 patients were included in the analysis. Patient demographics and ocular characteristics are listed in Table 1. A total of 309 eyes underwent phacoemulsification alone and 76 eyes underwent phaco-KDB. There were no statistically significant differences in age or sex between the two groups. In both cohorts, primary open angle glaucoma was the most common glaucoma subtype followed by pseudoexfoliation glaucoma and chronic angle closure glaucoma. Pre-operative visual acuity was better in glaucomatous patients undergoing phacoemulsification alone (*p* = 0.03). Axial length and keratometry values were similar between the two groups. All refractions took place 21–365 days after the surgery.

**Table 1:** Patient demographics and ocular characteristics of glaucoma patients undergoing cataract surgery with and without Kahook Dual Blade (KDB) goniotomy

	Glaucoma with KDB (*n* = 76)	Glaucoma without KDB (*n* = 309)	*p*-Value
Gender			
Male	28 (36.8%)	120 (38.8%)	
Female	48 (63.2%)	189 (61.2%)	0.79
Age, mean (SD) (years)	72.4 (8.3)	72.2 (10.4)	0.92
Type of Glaucoma			
POAG	61 (80.3%)	228 (73.8%)	Reference
CACG	2 (2.6%)	19 (6.2%)	0.38
PXF	11 (14.5%)	29 (9.4%)	0.42
Other	2 (2.6%)	33 (10.7%)	
Preoperative IOP (mmHg)	(n = 76)	(*n* = 304)	
Mean (SD)	15.5 (3.8)	14.5 (3.9)	
Median	15.0	14.0	0.05
Preoperative visual acuity (logMAR)			
Mean (SD)	0.254 (0.26)	0.394 (0.49)	
Median	0.176	0.301	0.03
Axial length (mm)	(*n* = 75)	(*n* = 302)	
Mean (SD)	24.3 (1.3)	24.3 (1.4)	
Median	23.9	24.2	0.84
Keratometry (D)	(n = 75)	(*n* = 305)	
Mean (SD)	44.0 (1.5)	44.0 (1.9)	
Median	43.8	44.0	0.64

*KDB* = Kahook Dual Blade, *IOP* = intraocular pressure, *POAG* = primary open angle glaucoma, *CACG* = chronic angle closure glaucoma, *PXF* = pseudoexfoliation, *SD* = standard deviation

**Fig. 1:**
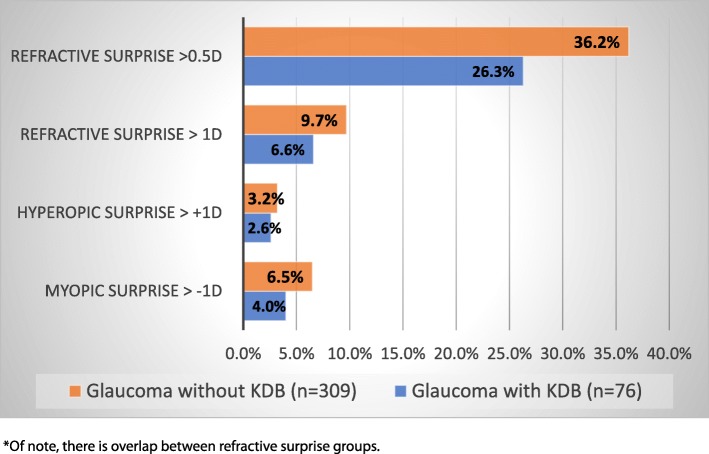
Refractive Surprise in Glaucoma Patients With and Without KDB (per eye)^*^

In glaucomatous eyes undergoing phaco-KDB, there were 20/76 (26.3%) cases of refractive surprise. The majority of refractive surprises in this group were between 0.5 and 1.0D of the intended target (*n* = 15), compared with only 5 cases being greater than ±1.0 D of the refractive target. In glaucomatous eyes undergoing phacoemulsification alone, there were 112/309 (36.2%) cases of refractive surprise with the majority falling between 0.5 and 1.0D of the intended target. There were 30 cases with greater than ±1.0D of refractive surprise in the glaucomatous eyes undergoing phacoemulsification alone. In both groups, myopic surprise was more common than hyperopic surprise as outlined in Fig. 1. There were no statistically significant differences between the two study groups using any definitions of refractive surprise.

As shown in Table 2, patients with refractive surprise greater than ±1.0 D (*n* = 35) had worse preoperative visual acuity (*p* = 0.01) and higher pre-operative IOP (*p* = 0.02) compared to those without refractive surprise of this amount (*n* = 350). Axial length and keratometry values were similar between those with and without refractive surprise. Further, the use of KDB at the time of phacoemulsification was not a statistically significant variable when comparing those with refractive surprise greater than ±1.0 D and those without refractive surprise (*p* = 0.40). For patients with refractive surprise greater than ±0.5 D, no ocular or patient characteristic was identified as a significant risk factor.

**Table 2:** Potential risk factors for refractive surprise in study patients

	Refractive Surprise (greater than ±1 D) N = 35	No Refractive Surprise N = 350	*p*-value
KDB			
Yes	5 (14.3%)	71 (20.3%)	
No	30 (85.7%)	279 (79.7%)	0.40
Preoperative visual acuity (logMAR)	(n = 35)	(n = 350)	
Mean (SD)	0.534 (0.60)	0.350 (0.44)	
Median	0.301	0.301	0.01
Preoperative IOP (mm Hg)	(n = 35)	(*n* = 345)	
Mean (SD)	16.4 (4.3)	14.5 (3.8)	
Median	16.0	14.0	0.02
IOP change (mm Hg)	(*n* = 25)	(*n* = 273)	
Mean (SD)	1.8 (6.8)	1.2 (4.3)	
Median	2.0	1.0	0.68
Axial length (mm)	(n = 35)	(*n* = 342)	
Mean (SD)	24.4 (1.4)	24.3 (1.4)	
Median	24.2	24.2	0.76
Axial length (mm)	(n = 35)	(n = 342)	
< 23.0 mm, n (%)	6 (17.1%)	53 (15.5%)	0.64
23.0–25.0 mm, n (%)	17 (48.6%)	190 (55.6%)	Ref
> 25.0 mm, n (%)	12 (34.3%)	99 (29.0%)	0.42
Keratometry (D)	(n = 34)	(*n* = 346)	
Mean (SD)	44.6 (3.3)	44.0 (1.6)	
Median	44.2	44.0	0.19

*KDB* = Kahook Dual Blade, *SD* = standard deviation, *mm* = millimeters, *D* = diopters

In a multivariate analysis of risk factors for refractive surprise greater than ±1.0 D, preoperative visual acuity had an adjusted odds ratio of 2.2 (95% CI: 1.5–3.3, *p* = 0.03) and pre-operative IOP had an adjusted odds ratio of 1.6 (0.8–3.5, *p* = 0.03). KDB goniotomy was not found to be a risk factor for refractive surprises greater than ±1.0 D, with an adjusted odds ratio of 0.5 (95% CI, 0.2–1.2, *p* = 0.32).

## Discussion

With advancing surgical techniques and improved pre-operative measurements and IOL calculations, today’s patient expects a predictable refractive outcome after cataract surgery. Prior studies have shown that glaucomatous patients undergoing phacoemulsification have higher rates of refractive surprise compared to patients without glaucoma [8]. Further, patients undergoing combination cataract and glaucoma procedures, whether filtering in nature or MIGS procedures, may be at increased risk for refractive surprise as well [16]. To our knowledge, our study is the first analysis of the effect of KDB goniotomy on refractive outcomes after cataract surgery. Our data demonstrate that the addition of KDB goniotomy at the time of cataract surgery does not change the rate of refractive surprise in patients with glaucoma.

Francis et al. and Yeh et al. have both demonstrated a higher rate of myopic surprise in patients with trabeculectomy performed prior to or at the time of cataract surgery. This refractive surprise has been attributed to the change in axial length that occurs in some patients with trabeculectomy. Traditionally, post-trabeculectomy, AL decreases as much as 0.91 mm at 12 months [11, 23]. This decrease in AL correlates with a reduction in IOP, with the largest decrease in AL occurring with an IOP below 9 mmHg [11]. Newer angle based and MIGS procedures typically produce less dramatic IOP lowering compared to trabeculectomy. One-year data show an average post-operative IOP after phacoemulsification with KDB goniotomy to be around 12 mmHg [19]. Since phacoemulsification combined with MIGS procedures may have a more physiologic IOP compared to filtering surgery, there is likely less of an impact on post-operative AL and thus, refractive outcomes.

Keratometry measurements also play a significant role in IOL calculations [15]. Because MIGS surgeries use the pre-existing clear corneal wound used for phacoemulsification, they should not add any induced astigmatism beyond what is already induced by the cataract extraction itself. This is in contrast to trabeculectomy, which increases with-the-rule astigmatism over time [24, 25].

While in our study there was no increase in refractive surprise related to AL or keratometry values, there was an increased risk of refractive surprise greater than ±1.0 D with worse pre-operative visual acuity and higher pre-operative IOP. The reasons for these associations are unclear. It is possible that eyes with worse pre-operative vision had worse ocular surface disease or increased lens density that resulted in less accurate IOL measurements. The higher incidence of refractive surprise in patients with higher pre-operative IOP could be related to a change in AL due to the IOP change although postoperative AL was not directly measured.

The effect on post-operative refraction has previously been evaluated in other MIGS procedures with mixed results. Kang et al. found no change in refractive outcome between phacoemulsification alone compared to phacoemulsification plus ECP [15]. Conversely, Sheybani et al. demonstrated that ECP performed in addition to phacoemulsification led to an increase in the rate of myopic surprise compared to cataract surgery alone [16]. A potential myopic result is not entirely surprising as ECP causes shrinkage and posterior rotation of the ciliary body, which could alter the effective lens position (ELP). UBMs performed after ECP have shown increased anterior chamber depth (ACD) as well which may correlate to a change in ELP [26]. While there is no published data comparing ACD and refractive outcome directly, prior studies have shown post-operative lens vault, which is reliant on ACD, is a potential reason for glaucoma patients experiencing post-operative refractive surprise [27, 28].

Surgically, the KDB goniotomy procedure is similar in nature to other angle procedures that incise or ablate the trabecular meshwork such as the Trabectome (Neomedix Corporation, Tustin, CA). Since they lower IOP through bypass of the trabecular meshwork and produce minimal change to surrounding structures, an anatomical shift in ELP or ACD would be unlikely after goniotomy. A study by Luebke et al. has supported this. They found no difference in refractive outcomes between patients who received cataract surgery alone and those who had cataract surgery with trabectome [14]. Similarly, our study found no difference in refractive surprise between patients who received cataract surgery with or without KDB goniotomy.

Limitations of this study include the retrospective nature and small sample size. Given this was a retrospective study, there was no randomization however patient and ocular demographics were similar between the two groups. In addition, the majority of the patients underwent phacoemulsification without KDB. Future studies should include larger sample sizes and directly investigate AL, keratometry, and ACD differences pre- and post-operatively and correlate them with the degree of IOP change.

## Conclusions

In conclusion, KDB goniotomy, when performed at the time of cataract surgery in patients with glaucoma does not change refractive outcomes. Specifically, higher pre-operative IOP and worse pre-operative visual acuity in glaucoma patients led to higher rates of refractive surprise regardless of whether KDB is performed in addition to phacoemulsification. Future prospective comparison studies of various angle-based and MIGS procedures and their effects on refractive outcome are warranted.

## Data Availability

The dataset analyzed during the current study may be available from the corresponding author on reasonable request.
